# Detecting CTP truncation artifacts in acute stroke imaging from the arterial input and the vascular output functions

**DOI:** 10.1371/journal.pone.0283610

**Published:** 2023-03-30

**Authors:** Ezequiel de la Rosa, Diana M. Sima, Jan S. Kirschke, Bjoern Menze, David Robben

**Affiliations:** 1 icometrix, Leuven, Belgium; 2 Department of Informatics, Technical University of Munich, Munich, Germany; 3 AIMS Laboratory, Center for Neurosciences, Vrije Universiteit Brussel, Brussels, Belgium; 4 Neuroradiology, School of Medicine, Technical University of Munich, Munich, Germany; 5 Department of Quantitative Biomedicine, University of Zurich, Zurich, Switzerland; 6 Medical Imaging Research Center (MIRC), KU Leuven, Leuven, Belgium; 7 Department of Electrical Engineering, Medical Image Computing (MIC), ESAT-PSI, KU Leuven, Leuven, Belgium; RKH Klinikum Ludwigsburg, GERMANY

## Abstract

**Background:**

Current guidelines for CT perfusion (CTP) in acute stroke suggest acquiring scans with a minimal duration of 60-70 s. But even then, CTP analysis can be affected by truncation artifacts. Conversely, shorter acquisitions are still widely used in clinical practice and may, sometimes, be sufficient to reliably estimate lesion volumes. We aim to devise an automatic method that detects scans affected by truncation artifacts.

**Methods:**

Shorter scan durations are simulated from the ISLES’18 dataset by consecutively removing the last CTP time-point until reaching a 10 s duration. For each truncated series, perfusion lesion volumes are quantified and used to label the series as *unreliable* if the lesion volumes considerably deviate from the original untruncated ones. Afterwards, nine features from the arterial input function (AIF) and the vascular output function (VOF) are derived and used to fit machine-learning models with the goal of detecting unreliably truncated scans. Methods are compared against a baseline classifier solely based on the scan duration, which is the current clinical standard. The ROC-AUC, precision-recall AUC and the F1-score are measured in a 5-fold cross-validation setting.

**Results:**

The best performing classifier obtained an ROC-AUC of 0.982, precision-recall AUC of 0.985 and F1-score of 0.938. The most important feature was the AIF_coverage_, measured as the time difference between the scan duration and the AIF peak. When using the AIF_coverage_ to build a single feature classifier, an ROC-AUC of 0.981, precision-recall AUC of 0.984 and F1-score of 0.932 were obtained. In comparison, the baseline classifier obtained an ROC-AUC of 0.954, precision-recall AUC of 0.958 and F1-Score of 0.875.

**Conclusions:**

Machine learning models fed with AIF and VOF features accurately detected unreliable stroke lesion measurements due to insufficient acquisition duration. The AIF_coverage_ was the most predictive feature of truncation and identified unreliable short scans almost as good as machine learning. We conclude that AIF/VOF based classifiers are more accurate than the scans’ duration for detecting truncation. These methods could be transferred to perfusion analysis software in order to increase the interpretability of CTP outputs.

## Introduction

Treatment decision making in acute ischemic stroke is mostly guided by computed tomography (CT) imaging, as the technique allows to answer (at least) four crucial questions regarding the patient brain’s condition: 1) Is there hemorrhage? 2) Is there any thrombus that could be targeted? 3) Is there already irreversibly damaged tissue (a.k.a. *core*)? 4) Is there salvageable tissue (a.k.a. *penumbra*, tissue at risk but potentially recoverable)? [1]. While the first two questions can be answered with non-contrast CT and CT angiography, respectively, the last two questions are typically addressed through CT perfusion (CTP). CTP is of major importance for neuroradiologists as it allows the identification of patients that could benefit from recanalization therapies [2]. In this context, distinguishing potentially salvageable brain tissue from already necrosed areas drive the therapheutical decision making.

In clinical routine, CTP post-processing software is used to estimate perfusion maps and to quantify perfusion lesion volumes. The perfusion maps used in acute ischemic stroke are derived from the CTP contrast attenuation curves and are cerebral blood volume, cerebral blood flow (CBF), mean transit time and time to the maximum of the residue function (Tmax). There exist several different techniques implemented in clinical and/or research software packages to estimate these perfusion metrics. Among the most widely used are the Fourier transform and the delay-invariant singular value decomposition deconvolution techniques using time-shift [3] or block-circulant approaches [4, 5]. Independently of their functioning, the end goal of CTP software packages is the accurate quantification of perfusion maps and, consequently, the reliable volumetric quantification of the brain lesions. Despite the vast adoption of CT perfusion software in clinical routine, there are well known and persistent pitfalls of these techniques that hamper the brain lesion quantification and hence their interpretation, as described in [6–9]. This work focuses on the so called *truncation* of the time attenuation curves, which could be defined as the early ending of the CTP acquisition that precludes the entire capture of the tissue perfusion phases [8].

CTP truncation artifacts have extensively been observed in previous works [10–17] and are related to several sources: *i*) the type of deconvolution used to process the CTP images, *ii*) the biological and physiological variability of the patients (e.g. patient size and the cardiac output alter the contrast delivery through the brain [16]), *iii*) physiopathological conditions that prolong the contrast-agent passage through the affected tissue, which happens in the hypoperfused tissue due to the ischemic occlusion [10, 13] or in patients with severe intracranial vascular narrowing or multiple intracranial emboli [6], and *iv*) the contrast injection and CTP acquisition protocols (e.g. contrast injection rate, the pre-contrast scanning duration, synchronization between contrast injection and acquisition, etc.).

As described in practical acute stroke imaging recommendations, the CTP analysis should include a quality control step that checks for complete acquisition of the perfusion curves including both the contrast agent wash-in and wash-out phases [8, 9, 18]. Visual identification of truncated AIF/VOF and/or time attenuation curves has been conducted in previous studies [14, 15]. Despite the fact that visual quality control could easily detect truncated perfusion curves, it is not straightforward to understand the implications of such curves truncations over the quantified lesion volumes. Thus, finding whether the truncation effects are strong enough to considerably perturb the quantified perfusion volumes could only be assessed through quantitative analyses. A major step in understanding the quantitative impact of truncation artifacts over the perfusion maps was done in [16]. The work showed that truncation artifacts depend on the truncation degree and affect the perfusion metrics differently depending on the used deconvolution algorithm. Moreover, the CTP truncation effects over the brain lesion volumes were studied in [17]. The authors found that a 60 second scan duration is enough to avoid volumetric errors in 95% of their analyzed scans. These results have later been adopted as a practical recommendation for the implementation of CTP in acute stroke [18]. In clinical routine, however, different centers or scanner operators make use of post-processing software from different vendors (and with diverse deconvolution algorithms), as well as different contrast injection and CTP acquisition protocols. Shorter acquisitions are frequently adopted by centers in order to reduce the exposure of the patient to ionizing radiation under the ALARA (i.e. as low as reasonably achievable) principle. Based on these considerations, it is possible that scans with shorter than 60 second scan duration could reliably estimate lesion volumes while scans with different characteristics could suffer from truncation errors even while having a 60–70 second acquisition duration.

In this work we propose a tool for the automatic identification of unreliable perfusion volumes due to insufficient scan duration. Our proposal makes use of simple and easy to extract features derived from the vascular perfusion curves (i.e. the arterial input function, AIF, and the vascular output function, VOF). Experiments on the public ISLES’18 dataset show that truncation artifacts impact the perfusion-derived features, hence allowing their identification with machine learning models. The proposed approach increases the interpretability of acute ischemic stroke outputs obtained in clinical practice with CTP post-processing software.

## Materials and methods

### Data

The ISLES’18 dataset is used for our experiments [19, 20]. The database is multi-center and multi-scanner and includes 156 CTP scans obtained from 103 acute stroke patients. For our experiments, we have used the preprocessed scans from the ISLES 2018 challenge (http://www.isles-challenge.org/). The CTP volumes have been motion corrected, coregistered and spatio-temporally resampled (256 × 256 matrix, 1 volume per second). A full dataset description can be found in [19].

### Simulating shorter CTP scans

We simulate shorter CTP scan durations by repeatedly discarding a 1 second timepoint from the end of the series until reaching the 10 first seconds of it. Note that the number of truncated simulated series varies from scan to scan, depending on its original total duration.

### CTP post-processing

Each truncated CTP series is analyzed using a research version of ico**brain cva** 1.4.1 (icometrix, Leuven, Belgium), an FDA-cleared and CE-marked software for acute stroke CTP post-processing. Each truncated series is processed using experts’ manually annotated vascular functions available in [21]. Please note that the manual AIF/VOF does not change location for all shorter versions of a same scan. The vascular functions from each truncated scan are retained for the subsequent experiments.

Perfusion maps (Tmax, CBF, cerebral blood volume and mean transit time) are obtained through delay-invariant singular value decomposition deconvolution. Absolute and relative CBF maps are computed, where the relative rCBF map is obtained after normalization of the absolute one using mean control tissue values. Control tissue is defined by the software as Tmax < 6s [22]. Quantification of the hypoperfused and core lesion volumes is automatically obtained by the software using Tmax > 6s [22] and rCBF < 0.38 (within the hypoperfused tissue area), respectively. The used rCBF cutoff (which is set in the software just for the purpose of these experiments) has been identified as optimal for the ISLES’18 dataset [19].

### Defining truncation artifacts

In order to label each shorter scan version as *reliable* or *unreliable* (i.e., considerably suffering from truncation artifacts), we first check that the original unshortened scan does not already suffer from truncation artifacts. As such, scans are labeled to be *stable* if truncation of the final 6 frames or less did not impact the computed volumes by more than 2.5 ml [17]; otherwise, scans are labelled as *unstable* ones. For our experiments, all unstable scans have been discarded from further analyses.

**Fig 1:**
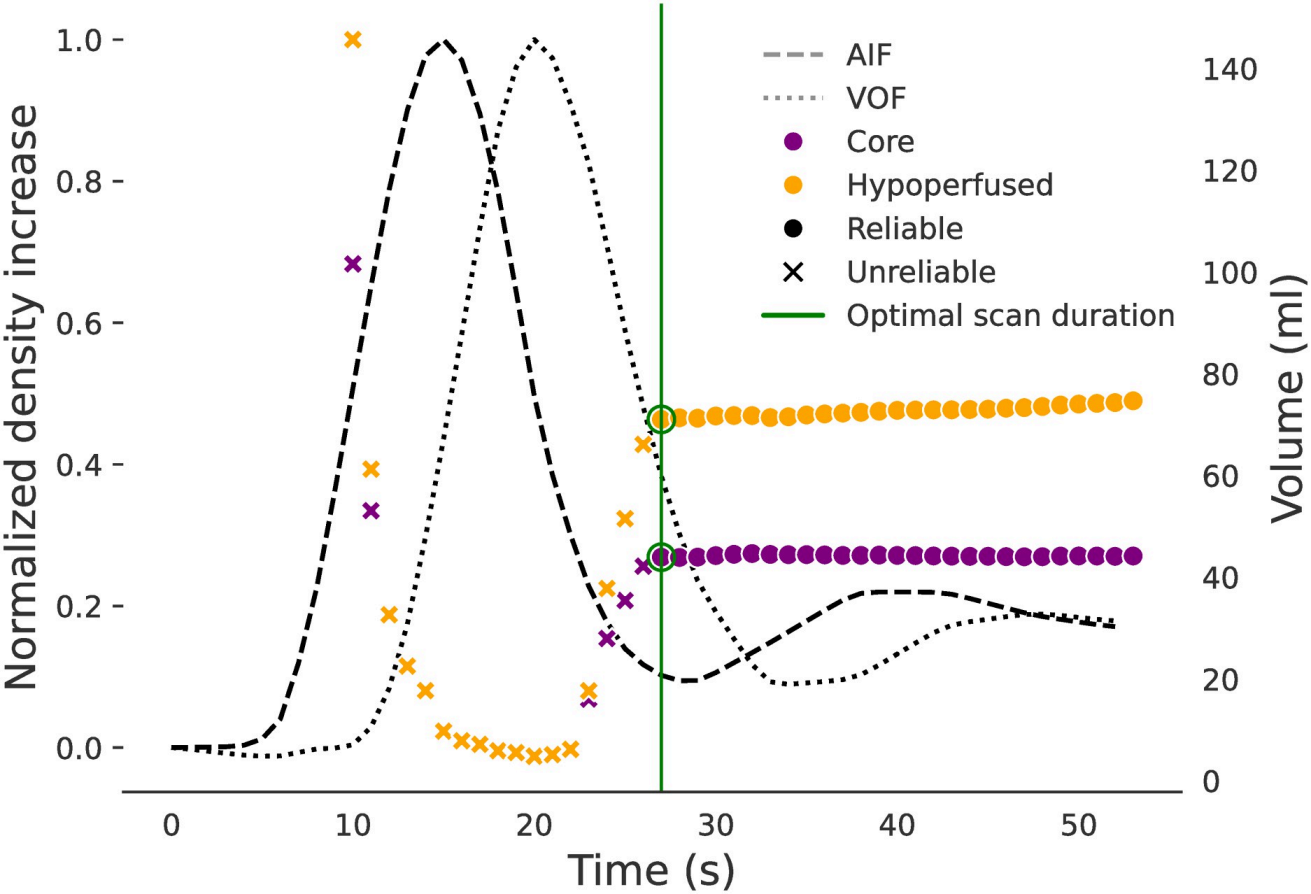
Reliable/unreliable lesion volumes computed at various scan durations. The arterial input function (AIF) and the vascular output function (VOF) are displayed as reference.

The truncated series from all stable scans are labelled as *reliable* if the corresponding hypoperfused and core volumes deviated < 10% or < 5 ml from the untruncated volume estimates. Otherwise, the truncated scan (and all its shorter versions) are labelled as *unreliable*. Scans with a stable hypoperfused lesion smaller than 5 ml are excluded from the analysis as their reliability can not be trusted. Besides, for each CTP series, the optimal scan duration (OSD) is defined as the shortest scan duration providing reliable volume estimates. Fig 1 shows a stable CTP scan example with its corresponding reliability truncation labels.

### Machine learning for CTP truncation detection

Machine learning algorithms have been widely used to assess the quality of medical images [23–25]. We explore different machine learning models that could detect unreliably truncated scans by solely using information extracted from the vascular functions. The benefits of using the AIF and VOF to detect truncation artifacts are two-fold. First, the perfusion curves are always available in this imaging modality. Second, as they cover the entire perfusion event (note that these curves represent the contrast concentration inlet and outlet to the brain), they contain rich information for the problem under study. Consequently, it is needed to extract meaningful perfusion features that are impacted by an insufficient scan duration and that are, also, predictive of the truncation artifacts. Those features should capture the perfusion phases of the contrast-agent wash-in and wash-out and, ideally, they should be unaltered by the different CTP protocols used in clinical routine.

#### Feature extraction

All the explored machine learning algorithms are fed with the following 9 AIF/VOF derived features:

- Scan duration
- AIF/VOF time to the peak of the function (argmax{AIF}, argmax{VOF})
- The *AIF/VOF coverage*, defined as the time difference between the peak of a signal and the scan duration:
  * AIF_coverage_ = scan duration—*argmax*{AIF}
  * VOF_coverage_ = scan duration—*argmax*{VOF}
- AIF/VOF upward and downward contrast increase
  * AIF_UCI_ = AIF_t=argmax{AIF}_—AIF_t=0_
  * AIF_DCI_ = AIF_t=argmax{AIF}_—AIF_t=scan duration_
  * VOF_UCI_ = VOF_t=argmax{VOF}_—VOF_t=0_
  * VOF_DCI_ = VOF_t=argmax{VOF}_—VOF_t=scan duration_

**Fig 2:**
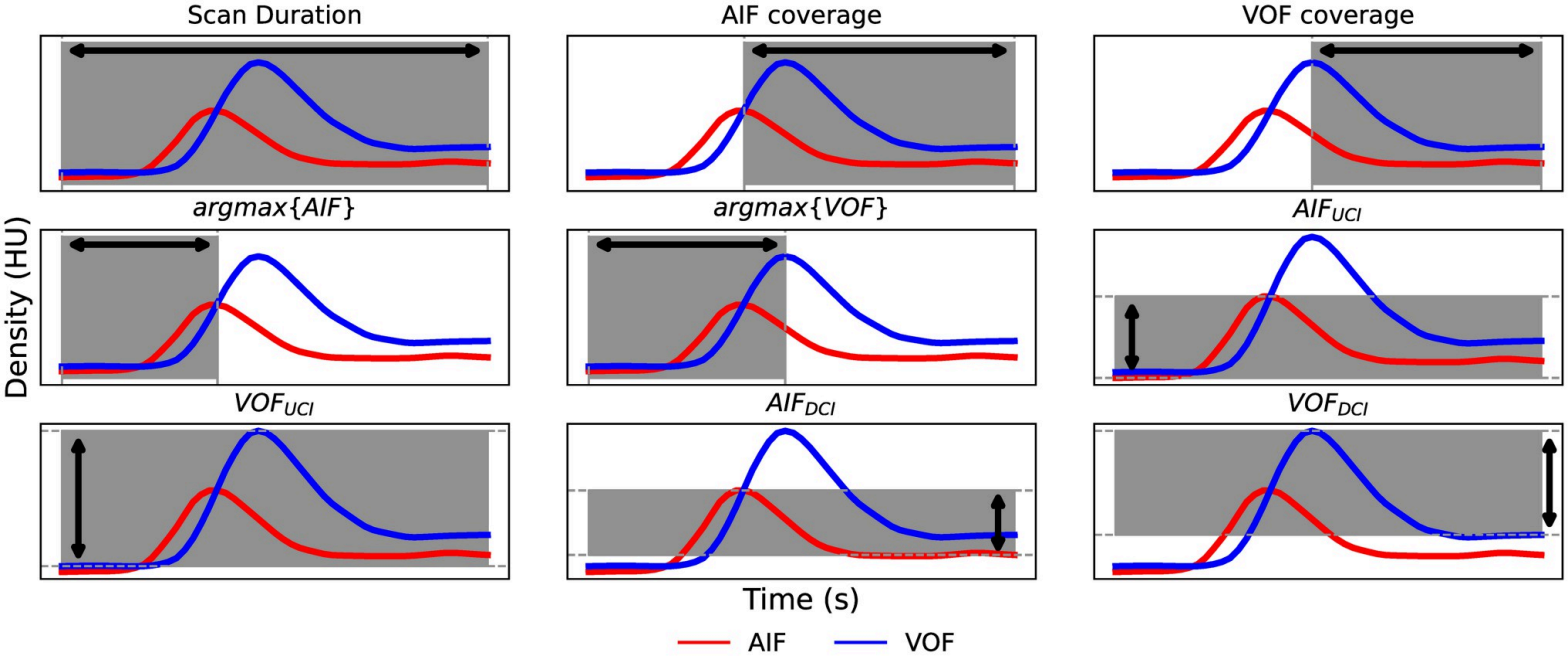
AIF and VOF derived features used to feed the machine learning algorithms. AIF: arterial input function; VOF: venous output function; HU: Hounsfield units; UCI: upward contrast increase; DCI: downward contrast increase.

All features are visually represented in Fig 2.

#### Classifiers & model fitting

We train six statistical/machine learning classifiers with the aim of detecting *reliable* and *unreliable* truncated scans. The trained models make use of linear or non-linear decision functions and are: *i*) random forests, *ii*) multivariate logistic-regression, *iii*) support vector machines with linear kernel, *iv*) support vector machines with radial basis kernel, *v*) Adaptive boosting (aka, Adaboost [26]) and *vi*) Gradient boosting [27]. In order to find the optimal set of parameters to fit a model, a Bayesian search is conducted by sampling over the parameter-space of each model and by evaluating its performance in a train set, 3-fold cross-validation setting. The final set of parameters chosen to parametrize the model is the one maximizing the area under the precision-recall curve (PR-AUC). All models are trained and optimized using the scikit-learn Python library [28].

#### Data augmentation

We augment the training sets by generating synthetic samples in order to model different perfusion scenarios, such as the variable pre-contrast agent duration and the variable contrast increases of the perfusion curves. Note that a different timing in the contrast bolus arrival alters the CTP scan duration but does not alter the presence of truncation artifacts. Likewise, the AIF and VOF contrast increase depends on the contrast agent iodine concentration. However, as the deconvolution algorithm is independent from the AIF/VOF absolute amplitudes, a variable vascular contrast increase does not alter the presence of truncation artifacts.

Simulation of contrast injection protocol variations is conducted by perfusion-specific data augmentation as similarly done in [21, 29]. Uniform distributions are used to randomly modify the pre-contrast agent duration and vascular contrast increases. When simulating variable pre-contrast duration, pre-contrast timing dependent features are increased or decreased by the same random factor (argmax{AIF}, argmax{VOF} and *scan duration*). For modelling variable contrast increases, the features AIF_UCI_, AIF_DCI_, VOF_UCI_ and VOF_DCI_ are scaled by a random factor.

#### Experiments

We perform a 5-fold cross-validation experiment using an 80–20% train-test data split. The data splitting is conducted at the *scans* level, assuring that *i*) all untruncated and truncated versions of a same scan belong to the same fold and *ii*) the same data-splits are used to fit all the considered models. Only the training data is used parametrise the models and to select the classifiers’ operating point. Truncation predictions are later inferred over the unseen test data.

Besides, we compare the machine learning models against a baseline classifier which solely uses the *scan duration* as discriminant-rule. The classifier *g* operates as follows:

$$\begin{array}{c} g\left(\text{scan}\;\text{duration},\theta \right)=\{\begin{array}{cc} reliable & \text{if}\;\text{scan}\;\text{duration}\;>=\;\theta \;\\ unreliable & \text{if}\;\text{scan}\;\text{duration}\;<\;\theta \; \end{array} \end{array}$$ (1)

with *θ* a scan duration cutoff. This baseline is motivated by the CTP guidelines, which only consider the duration of a scan to avoid truncation artifacts during CTP acquisition [18]. Specifically, these guidelines suggest a cutoff of *θ* = 60 seconds in Eq 1.

In order to understand the relevance of the AIF-VOF extracted features to discriminate *reliably* and *unreliably* truncated scans, we conduct a bootstrapping experiment by resampling 100 times the original database. In each iteration, a sample was drawn with replacement and was used to fit a classifier as described in Section *Classifiers & Model Fitting*. The relative feature importance is measured as defined in [27] for decision tree ensembles. Briefly, the feature importance is calculated at the classifiers’ tree level as the impurity decay across all the nodes where that feature was used to create a split [30]. The final feature importance is computed as the average feature importance over all the considered trees. The mean and standard deviation feature importance for all features are reported. The chosen classifier for this experiment is the best performing one in terms of precision-recall AUC.

#### Performance evaluation

The mean, standard deviation, 5th-95th percentiles and minimum and maximum of the scan duration and the optimal scan duration are reported for the entire dataset. The different algorithms’ performance are evaluated by conducting ROC and precision-recall (PR) analysis. The area under the ROC and precision-recall curves are used as general classifier performance metrics. Besides, we measure the binary classification performance at the operating point closest to an ideal classifier with *precision = recall = 1*. The operating point is chosen from the fitted classifier as $argmin\{\sqrt{{\left(precision_{t}-1\right)}^{2}+{\left(recall_{t}-1\right)}^{2}}\}$, with *t* different classifier thresholds. Performance is measured in terms of precision ($\frac{TP}{TP+FP}$), recall ($\frac{TP}{TP+FN}$) and F1-score ($\frac{2*TP}{2*TP+FP+FN}$), where acronyms represent TP: true positives, TN: true negatives, FP: false positives and FN: false negatives. The same binary classification metrics are reported for our baseline scan duration classifier, by making use of cutoffs *θ* = [30, 40, 50, 60] *s*. For these defined metrics, an *unreliable* truncation sample is considered as positive and a *reliable* truncation sample as negative.

## Results & discussion

From the 156 analyzed scans, 123 scans (78.8%) are retained for further analysis. The remaining scans are discarded since 18 (11.5%) are unstable, 14 (8.9%) have hypo-perfused volumes < 5 ml or are free from CTP lesions, and 1 scan (0.6%) is corrupted by motion artifacts. A total of 4621 synthetically truncated scans are obtained from the retained stable cases, from which 2353 (50.9%) are labelled as *reliable* and 2268 (49.1%) as *unreliable*.

Descriptive statistics about the optimal scan duration are summarized in Table 1. It can be appreciated that ∼ 40 s scan duration suffices to get accurate perfusion volumes in 95% of the dataset and 43 s scan duration avoids truncation in the entire ISLES’18 dataset. At first sight these OSD values might seem much shorter than the 60-second recommended duration in the CTP guidelines [18]. However, the used ISLES’18 dataset has very short pre-contrast acquisitions that does not always reach the guidelines’ recommended 5–10 s. Note that the median AIF peak (i.e. argmax{AIF}) across all scans is 15.6 ± 4.5 s, with a minimum AIF peak at 5 s. Thus, some ISLES’18 scans have no pre-contrast acquisition at all, as seen in S1 Fig. Therefore, compensating for the short pre-contrast duration would increase the 95th percentile OSD value of Table 1 to a ∼ 45–50 s scan acquisition. This result is in line with earlier research: it was shown that in scans with 10 s of pre-contrast duration a ∼ 53 s acquisition is needed to get reliable perfusion volumes in 90% of the scans [17].

**Table 1:** Descriptive statistics of the stable CTP scans. SD: Scan duration; OSD: Optimal scan duration. Std: standard deviation; P: percentile. AIF: arterial input function. All metrics are reported in seconds.

	SD	OSD	(OSD—argmax{AIF})
Mean (std)	46.6 (5.3)	28.4 (5.7)	12.9 (3.7)
(Min, Max)	(31.0, 64.0)	(19.0, 43.0)	(8.0, 30.0)
(P5^*th*^, P95^*th*^)	(43.0, 60.2)	(20.0, 39.8)	(10.0, 19.9)

The optimal scan duration reported in Table 1 depends on the pre-contrast scan duration, which is not standardized in clinical practice. In order to have a more informative metric less biased by the different CTP protocols, we compute the time difference between the OSD and the AIF peak (argmax{AIF}). In Table 1 this metric is reported for our entire database. Results show that on average our scans require ∼ 13 s following the AIF peak to obtain reliable perfusion volumes. In [17] the median OSD for scans with a 10 s baseline is ∼ 33 s. Let’s assume an average AIF peak of ∼ 15–20 s in a standard CTP acquisition protocol. Then ∼ 13–18 s following the AIF peak are needed in [17] to get reliable volumes in 50% of their scans. Thus, these results are comparable to our finding of ∼ 13 s on average following the AIF peak to get reliable volumes.

These OSD analyses of ISLES’18 show that an acquisition protocol using 60–70 s scan duration, with 5–10 s of pre-contrast acquisition, avoids truncation errors in the whole analyzed dataset. Thus, supporting the recommended scan duration of the CTP guidelines. Depending on the patients’ physiology, the contrast injection and/or the CTP acquisition protocols, even shorter acquisitions may, sometimes, reliably quantify CTP lesion volumes. However, from a risk-benefit perspective it is strongly inadvisable to shorten CTP scan durations since *i*) it is not possible to know *a-priori* the OSD needed for a particular patient/scan, and *ii*) the risk of inaccurately estimating the lesion volumes due to a short acquisition is significantly larger than exposing the patient to an additional radiation exposure. Thus, an unreliable estimation of the core-penumbra mismatch may lead to a change in the treatment decision, which may have a drastic impact on the patient’s outcome. In a different scenario, an insufficient CTP acquisition may lead to the full re-scanning of the patient, thus significantly increasing the exposure to ionizing radiation and to the iodine contrast and, ultimately, delaying the treatment of the patient.

### Effect of the AIF choice

In order to understand the impact of different AIFs over the computed optimal scan duration, an inter-rater analysis is performed. In this experiment, we simulate truncation artifacts as described in section *Simulating Shorter CTP Scans* but using vascular functions selected by a different expert. The used annotations are the ones available in [21] and labelled in the work as *Rater #2*.

**Fig 3:**
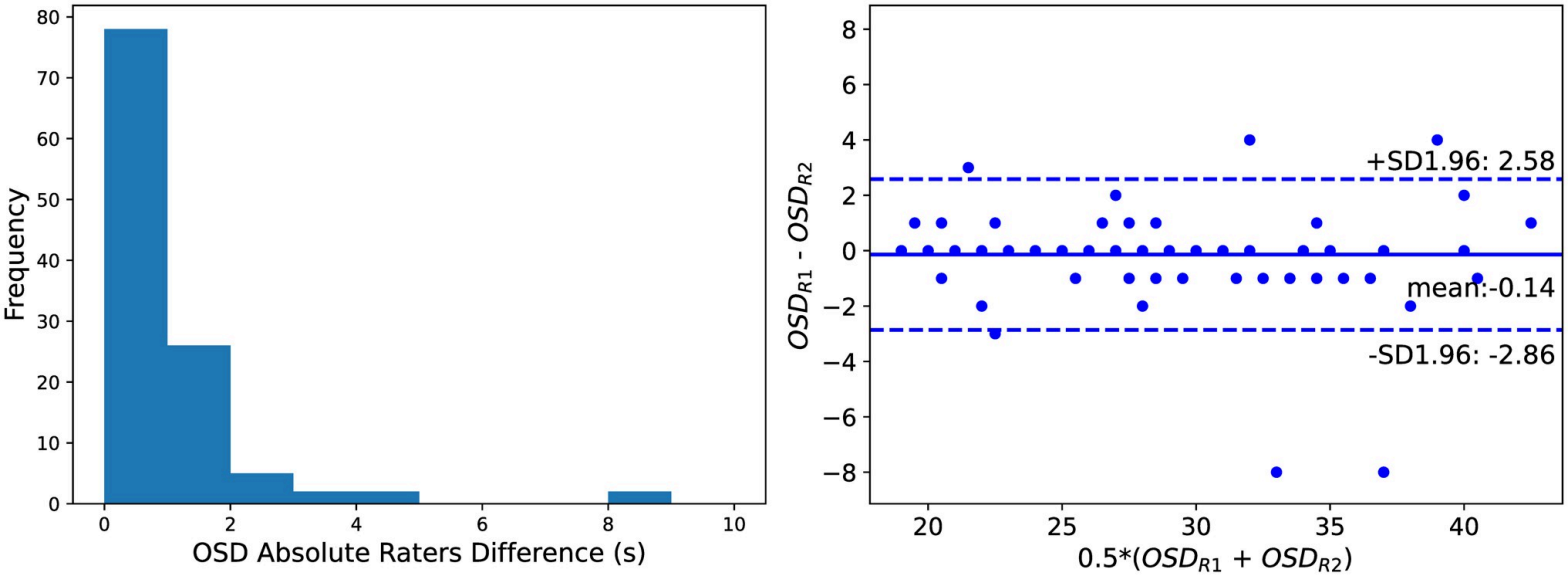
Optimal scan duration (OSD) inter-rater analysis. Left: Histogram of the OSD absolute differences between raters. Right: Bland-Altman plot of the OSD values for the raters. R1: Rater #1. R2: Rater #2.

In Fig 3 a histogram and a Bland-Altman plot of the inter-rater OSD values are shown (left and right figures, respectively). The majority of the scans (*n* = 78, ∼ 64%) show no time differences in the OSD obtained with vascular functions selected by the two raters. The 5^*th*^ and 95^*th*^ percentiles of the absolute OSD differences are respectively 0 s and 2.30 s. The maximum OSD difference between the raters is 8 s.

### Truncation artifacts detection

**Fig 4:**
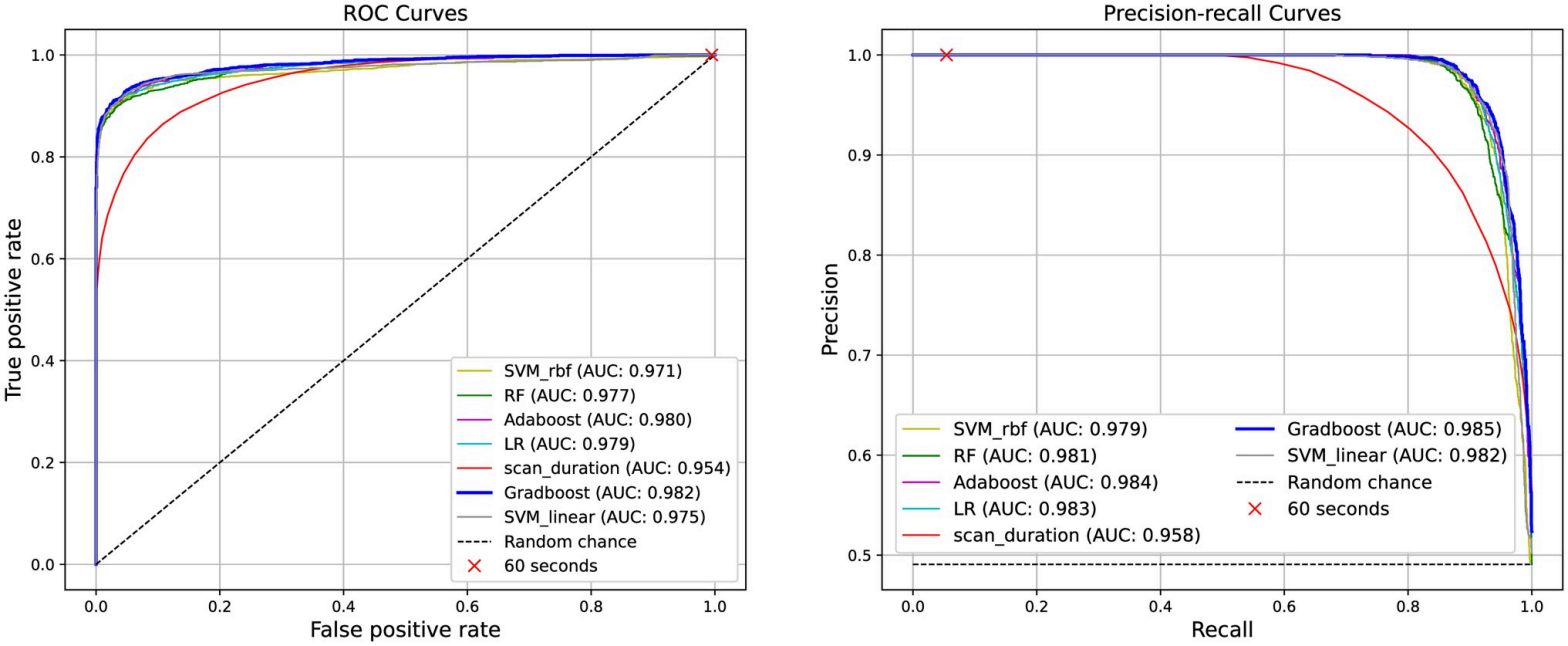
Receiver operating characteristic (left) and precision-recall (right) curves. AUC: area under the curve; SVM_linear: support-vector machine with linear kernel; SVM_rbf: support-vector machine with radial basis function kernel; RF: Random forests; LR: Logistic-regression; Adaboost: Adaptive boosting; Gradboost: Gradient boosting.

Fig 4 shows the ROC and precision-recall curves obtained with the different classifiers when differentiating *reliable* from *unreliable* truncated acquisitions. Overall it can be seen that classifiers yielded a similar high performance for both the considered metrics. The gradient boosting algorithm outperformed the remaining classifiers yielding an ROC-AUC of 0.982 and a precision-recall AUC of 0.985. When assessing the classifiers capability for detecting truncation artifacts at the chosen operating point, the results of Table 2 are obtained. The Gradient boosting method obtained the highest performance for detecting unreliable perfusion volumes (F1-Score = 0.938). All machine learning models have considerably outperformed the baseline classifier *g* in terms of ROC-AUC, PR-AUC and F1-Score (Table 2).

**Table 2:** Classifiers’ performance for detecting truncation artifacts. The used operating points are *θ* = [27, 30, 40, 50, 60] *s* for the baseline classifier *g*. Note that 27 s is the *optimal operating point* for *g*, defined as the closest point to the ideal classifier with *precision = recall = 1*. Reported metrics for the machine learning approaches are obtained at the optimal operating points. Outperforming values for each metric are shown in bold. SVM_Linear_: support-vector machine with linear kernel; SVM_RBF_: support-vector machine with radial basis function kernel; RF: Random forests; LR: Logistic regression; Gradboost: Gradient boosting classifier. Baseline classifier: threshold on scan duration.

Classifier	ROC-AUC	PR-AUC	Recall	Precision	F1-Score
SVM_Linear_	0,975	0,982	0,929	0,942	0,9305
SVM_RBF_	0,971	0,979	0,918	0,942	0,930
RF	0,977	0,981	0,898	**0,964**	0,929
Adaboost	0,980	0,984	0,922	0,945	0,934
LR	0,979	0,983	0,926	0,936	0,931
**Gradboost**	**0,982**	**0,985**	0,930	0,946	**0,938**
Baseline classifier	0,954	0,958	[0,885	0,864	0,875]_*θ*=27*s*_
			[0,926	0,813	0,866]_*θ*=30*s*_
			[0,997	0,597	0,747]_*θ*=40*s*_
			[**1,000**	0,503	0,670]_*θ*=50*s*_
			[**1,000**	0,493	0,660]_*θ*=60*s*_

For the baseline classifier *g*, the highest detection performance is obtained at the *optimal operating point θ* = 27 s (F1-score = 0.875). When using the standard cutoff *θ* = 60 s, the baseline classifier showed the maximal recall of 1.0 with a low precision of 0.493 and low F1-score of 0.660. These results are expected as all the analyzed scans have OSD values much lower than 60 s (Table 1). The ROC and precision-recall operating points at *θ* = 60 s are shown in Fig 4. It can be appreciated that this operating point falls on the boundaries of the classifiers’ ROC and precision-recall curves. These results suggest that using a 60 s scan duration is a safe and needed recommendation for acquiring CTP images, but it is a poor criterion in order to identify truncation errors at CTP post-processing stages.

#### Effect of the data augmentation

We evaluated the impact of the perfusion specific data augmentation strategy over the best performing machine learning model. For this experiment, a Gradient boosting classifier is trained using the original, un-augmented dataset as described in the section *Classifiers & Model Fitting*. While an ROC-AUC = 0.982, PR-AUC = 0.985 and F1-Score = 0.938 are obtained when using data augmentation, an ROC-AUC = 0.980, PR-AUC = 0.983 and F1-Score = 0.933 are obtained when training the model without augmenting the dataset. Our results show that simulating different perfusion scenarios (namely, variable contrast-increases and variable bolus arrival times) improves the model’s performance.

### Importance of the AIF and VOF features

**Fig 5:**
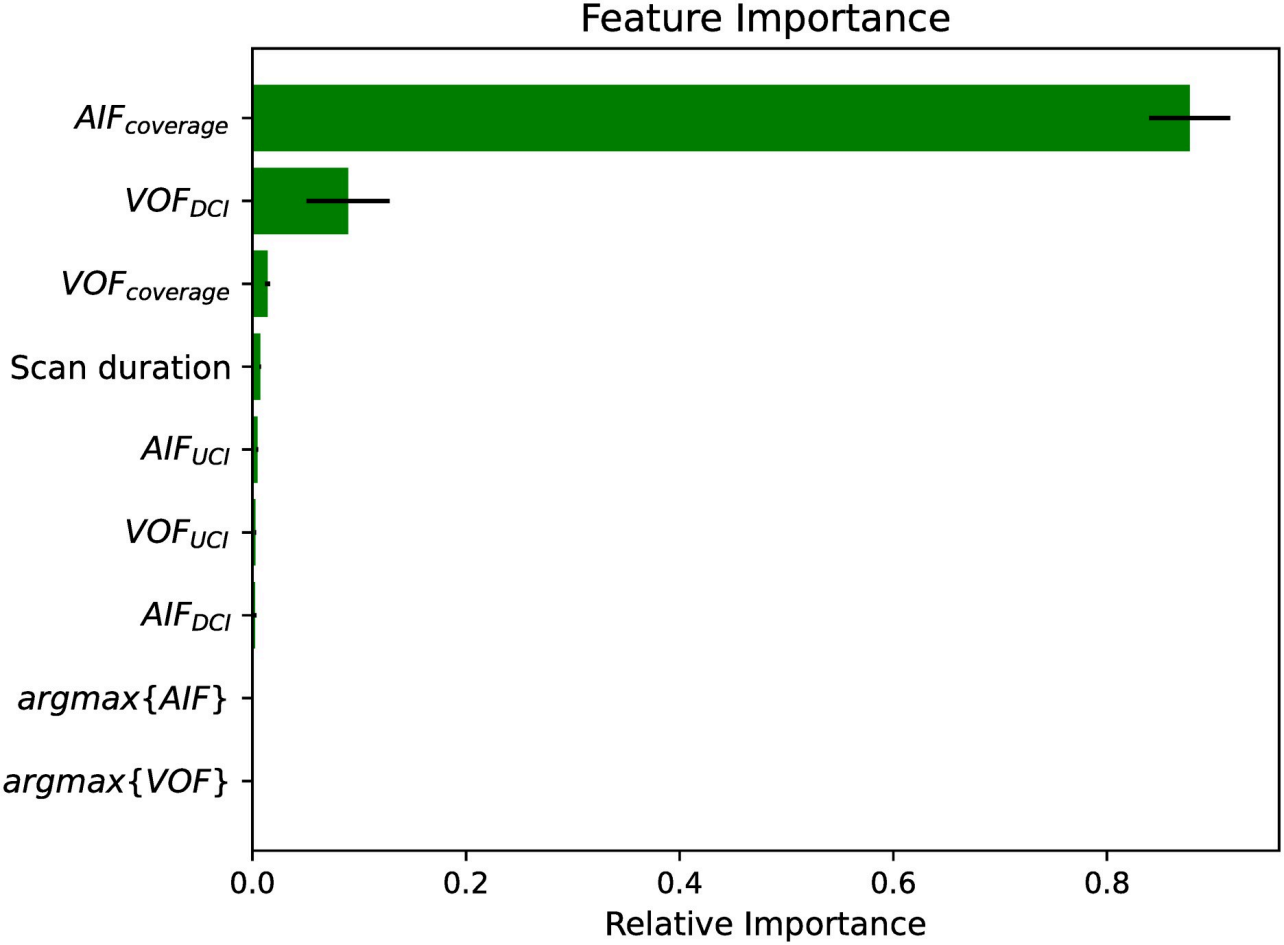
Relative feature importance for 100 bootstraps with a Gradient boosting classifier. Bars (error-bars) represent mean (standard deviation). AIF: arterial input function; VOF: venous output function; UCI: upward contrast increase; DCI: downward contrast increase.

Fig 5 summarizes the different features’ relevance obtained when fitting 100 Gradient boosting classifiers in a resampling with replacement bootstrapping fashion. The AIF_coverage_ shows to be the most crucial feature for detecting *unreliable* perfusion volumes due to truncated acquisitions. Besides, the VOF_DCI_ and the VOF_coverage_ also result to be important features for the machine learning model. The large predictive value of the AIF_coverage_ and the VOF_coverage_ features can be related to their robustness to variable pre-contrast agent duration. The *scan duration* feature, instead, is affected by the CTP acquisition protocols and as such, shows slightly less relevance for the fitted models.

**Fig 6:**
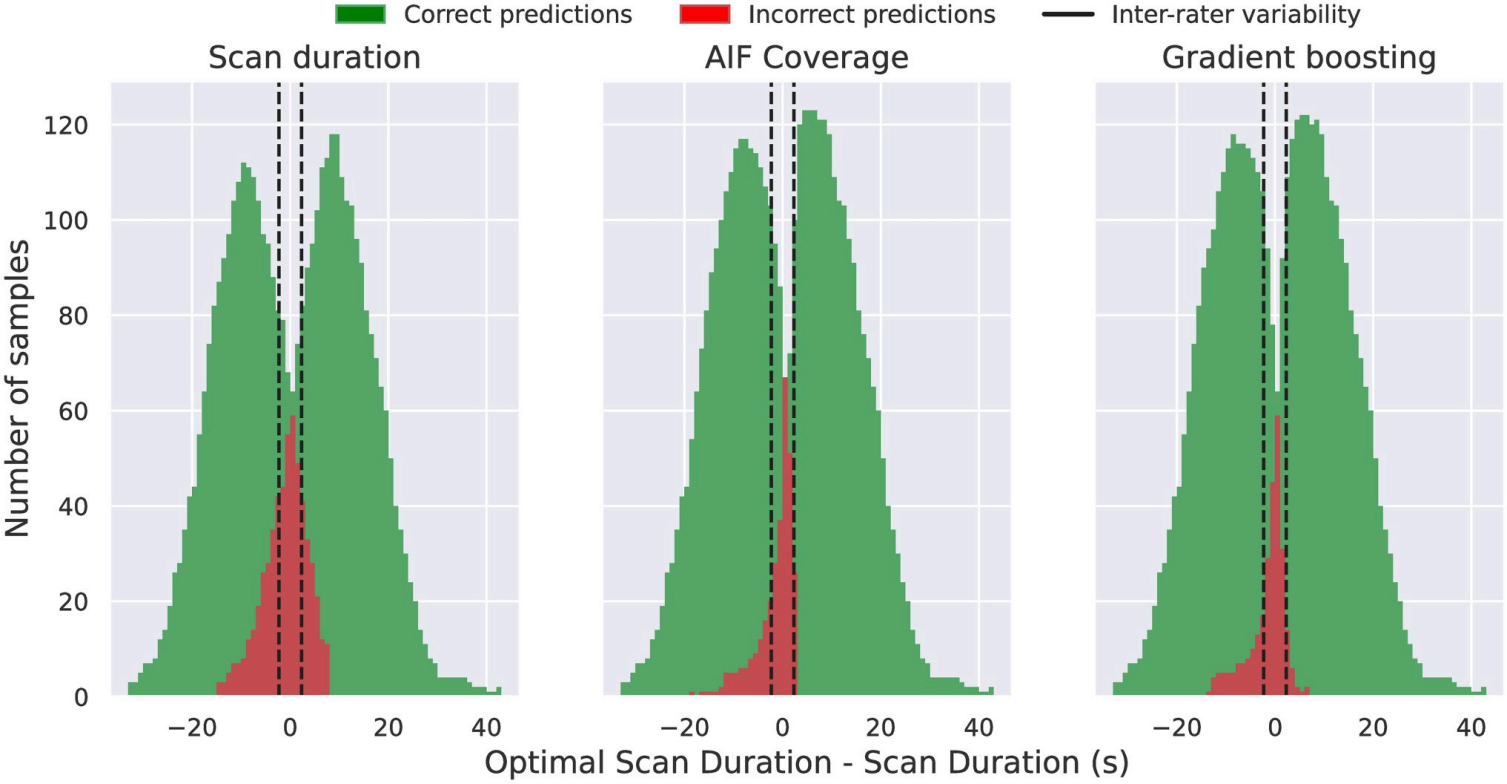
Histograms showing the difference between optimal scan duration and the scan duration for each classifiers’ predicted samples. *Correct predictions* comprises scans properly labelled as reliable or unreliable. *Incorrect predictions* comprises samples wrongly labelled as reliable or unreliable. Inter-rater variability lines are drawn at the 95% inter-rater values (± 2.30 s).

We also explore for each single AIF/VOF feature its discriminant power to detect truncation artifacts. To this end, we generate new classifiers *g*′ that operate as described in Eq 1 by using the considered AIF/VOF feature instead of the *scan duration* feature. The selection of the operating points and the validation of the classifiers are performed with the same criteria described in section *Performance evaluation* for the machine-learning models. In Table 3 the detection performance metrics achieved with the different feature classifiers *g*′ are summarized. It is worth noting that the top-ranked features in the bootstrapping experiment (namely AIF_coverage_, VOF_DCI_ and, VOF_coverage_, see Fig 6) are the ones achieving the highest discriminant performance for detecting truncation artifacts. The best classifier *g*′(*AIF*_*coverage*_) yielded a much better performance than the baseline classifier *g*(scan duration) (Tables 2 and 3). These results evidence that AIF_coverage_ is a strong discriminant feature for detecting truncation artifacts. Similar to our results [17], has observed that the VOF_coverage_ is an important feature impacted by truncation artifacts. Although AIF_coverage_ and VOF_coverage_ are strongly correlated features, our experiments show that AIF_coverage_ carries more truncation predictability than VOF_coverage_ (Table 3). The reason for this finding is that in severely truncated CTP series where the scan acquisition does not reach the VOF peak but it does reach the AIF peak, only the AIF_coverage_ can detect a truncation, as the VOF_coverage_ feature can not be reliably estimated. However, in cases where the CTP acquisition reaches the VOF signal peak, both the AIF_coverage_ and VOF_coverage_ carry similar truncation predictability. We have confirmed this statement after discarding very short CTP scans (e.g. with scan durations lesser than 15 and 20 s) from the database and after re-measuring the single features detection performance. The obtained ROC-AUCs for the AIF and VOF coverages were, respectively, 0.972 and 0.962 when discarding scans with durations shorter than 15 s, and 0.955 and 0.952 when discarding scans shorter than 20 s. Our results showed that the performance agreement between AIF_coverage_ and VOF_coverage_ increased while discarding short CTP series that did not cover the VOF signal peak. In clinical routine, a short duration scan not covering the AIF peak would lead to completely unreliable perfusion volumes and, as such, would hardly be used for treatment decision making. However, in practice it is still common to find CTP scans where the VOF peak has not been reached. Hence, we can conclude that both the AIF_coverage_ and VOF_coverage_ are features with good overall truncation predictability, though the AIF_coverage_ is a more robust feature as it works in a wider range of CTP truncation scenarios.

**Table 3:** Single features’ classification performance. The used cutoff is always the *optimal operating point*, defined as the closest point to the ideal classifier with *precision = recall = 1*. AIF: arterial input function; VOF: venous output function; UCI: upward contrast increase; DCI: downward contrast increase; ROC: receiver operating characteristic curve; PR: precision-recall curve; AUC: area under the curve.

Feature	ROC-AUC	PR-AUC	Precision	Recall	F1-score
AIF_DCI_	0.842	0.879	0.815	0.724	0.767
AIF_UCI_	0.597	0.631	0.571	0.465	0.513
*argmax*{*AIF*}	0.509	0.525	0.502	0.484	0.493
*argmax*{*VOF*}	0.693	0.752	0.649	0.592	0.619
**AIF**_**coverage**_	**0.981**	**0.984**	**0.936**	**0.928**	**0.932**
VOF_coverage_	0.956	0.962	0.874	0.880	0.877
VOF_DCI_	0.958	0.969	0.929	0.901	0.915
VOF_UCI_	0.765	0.816	0.765	0.597	0.671

### Scan duration, AIF coverage or machine-learning?

We compare the three classifiers outlined in this work: the baseline classifier *g* using the *scan duration*, the classifier *g*′ based on the *AIF coverage* and the Gradient boosting classifier which uses multiple AIF and VOF features. It is evident that both the AIF coverage *g*′ and the Gradient-boosting classifier considerably outperform the baseline approach *g* based on the scan duration. However, when comparing the Gradient boosting method with *g*′, a marginal improvement in performance is found (as seen from Tables 2 and 3). Moreover, the study of the feature’s importance has shown that the Gradient boosting method predicts outputs mostly using the AIF_coverage_ feature, together with some other features as VOF_DCI_ and VOF_coverage_ (see Fig 2). Thus, it is valid asking whether there is any benefit of using machine learning over a (simpler) classifier as *g*′.

In order to address this question we evaluate, for the three considered models, the distribution of the predicted samples in terms of their scan duration to optimal scan duration difference, as shown in Fig 6. While *g* misclassifies samples in a [-15, 8] s vicinity of OSD, *g*′ and the Gradient boosting classifier do it in a vicinity of [-19, 3] s and [-14, 8] s, respectively. Thus, within these temporal windows the classifiers struggled the most to correctly detect reliable/unreliable volumes and, outside this temporal window, the classifiers correctly predicted all samples. At their optimal operating points, all the three models are more accurate predicting truncation over very short scans (i.e. OSD > > scan duration) rather than over long duration ones (OSD < < scan duration). Besides, the closer a scan duration is to its OSD, the harder for the models is to correctly classify a sample. It is worth to point out that *g*′ and the Gradient boosting classifier generate most of the mis-classifications within the inter-rater OSD variability, a ‘gray zone’ interval where the *reliable* and *unreliable* labels suffer from larger uncertainty. With the aim of measuring the method’s performance over AIF-choice unbiased samples, we have quantified the classifiers’ error rates ($100\times \frac{\#\;\text{incorrect}\;\text{samples}}{\#\;\text{all}\;\text{samples}}$) outside the inter-rater variability range ([-2.30, 2.30] s). While *g* yielded an error rate outside the inter-rater interval of 7.8% (n = 314 misclassified samples), *g*′ and the Gradient boosting method yielded, respectively, a 2.5% (n = 102 misclassifications) and 2.4% (n = 98 misclassifications) error rates.

Our results show that both the Gradient boosting and the AIF_coverage_ based classifiers provide a good overall truncation predictability and consistently outperform the scan duration based approach. These models could increase the interpretability of CTP post-processing software by warning clinicians about potentially misleading and/or unreliable perfusion lesion volumes (i.e., volumetric errors greater than 10% or greater than 5 ml), which should be taken into account during treatment decision making. Additionally, such methods help identify reliable perfusion lesion measurements in scans not reaching the recommended 60–70 s acquisition. Considering their implementation in clinical routine CTP deconvolution packages, the AIF_coverage_ classifier might be preferred as *i*) it is more robust to the variable scan quality than the multi-feature machine learning (i.e., it requires measuring an always available and easy to extract feature, the AIF peak) *ii*) its implementation is straightforward (the model is free from hyper-parameters fitting) and *iii*) it has the advantage of being interpretable by radiologists and neurointerventionalists.

### Limitations and future directions

There are some considerations about this research that should be cautiously taken. Firstly, there is no consistent definition for a scan to be *reliable* and, as such, its definition is somehow arbitrary. In this work, in order to define *reliability* we have adopted a quantitative criterion based on the perfusion volumes as similarly done in [17]. Alternative definitions of scan reliability could be based on changes on the treatment eligibility criteria used in the DAWN or DEFUSE-3 trials [31, 32]. An advantage of using a volumetry based criterion over a treatment eligibility one is the capability to identify subtle lesion changes due to inaccurate perfusion measurements that may not impact the treatment decision (e.g. in patients with a large perfusion lesion mismatch).

Secondly, it is worth to mention that our conclusions only hold for CTP analysis using time-invariant singular value decomposition deconvolution. Other techniques used for perfusion analysis might behave differently under truncation scenarios. Still, the delay-invariant singular value decomposition deconvolution is the most widespread and used algorithm in software packages [8, 33, 34]. Readers interested in the effect of CTP truncation over different parameter map estimation methods are referred to the work of [16], as such inter-algorithm comparisons are out of the scope of this research. It is worth saying that while the devised models only hold for the ISLES’18 database characteristics and for the deconvolution algorithm used in this study, the extracted features are generalizable and allow the adaptation of these models to other deconvolution algorithms or imaging modalities (as perfusion MRI).

Thirdly, the deployment of a truncation artifacts detection method in automatic CTP evaluation software is limited to the AIF/VOF selection performance. In this work, all the experiments have been conducted using manually annotated vascular functions. As such, failures in the CTP curves selection could produce a misleading truncation analysis using our proposed methodology. Nonetheless, recent approaches using dedicated artificial intelligence methods show efficacy and robustness to select vascular functions even under low quality CTP scenarios [21, 35].

Finally, future directions for this work might involve the machine-learning prediction of missing CTP time-points at the end of the series. As such, reconstructing the ending perfusion phase of the vascular functions could help improve the detection of truncation artifacts.

## Conclusion

We observe that acquiring 60 s CTP scans is sufficient to avoid truncation artifacts in the entire multi-center/scanner ISLES’18 dataset. However, at CTP post-processing stages, using the scans’ duration to detect truncation errors is sub-optimal. Depending on the patients’ physiology, the contrast injection and/or the CTP acquisition protocols, even shorter acquisitions may sometimes provide reliable lesion volumes. In order to identify unreliable short scans we extract AIF and VOF features that are impacted by truncation errors. These features are simple, robust to extract even in low quality acquisitions and are independent from the contrast injection and CTP acquisition protocols. The AIF_coverage_ proves to be the most predictive feature of truncation. Furthermore, when training classifiers with AIF/VOF derived features a high truncation detection performance is obtained. We conclude that these methods could be transferred to perfusion analysis software and may increase the interpretability of CTP outputs.

## Supporting information

S1 Fig Example case of an ISLES’18 scan with no pre-contrast acquisition. AIF: Arterial input function; VOF: Venous output function.
(TIF)

## References

[1] Konstas A, Goldmakher G, Lee TY, Lev M. Theoretic basis and technical implementations of CT perfusion in acute ischemic stroke, part 1: theoretic basis. American Journal of Neuroradiology. 2009;30(4):662–668. doi: 10.3174/ajnr.A1487 19270105 PMC7051780

[2] Albers GW, Goyal M, Jahan R, Bonafe A, Diener HC, Levy EI, et al. Ischemic core and hypoperfusion volumes predict infarct size in SWIFT PRIME. Annals of neurology. 2016;79(1):76–89. doi: 10.1002/ana.24543 26476022

[3] Smith M, Lu H, Trochet S, Frayne R. Removing the effect of SVD algorithmic artifacts present in quantitative MR perfusion studies. Magnetic Resonance in Medicine: An Official Journal of the International Society for Magnetic Resonance in Medicine. 2004;51(3):631–634. doi: 10.1002/mrm.20006 15004809

[4] Wu O, Østergaard L, Koroshetz WJ, Schwamm LH, O’Donnell J, Schaefer PW, et al. Effects of tracer arrival time on flow estimates in MR perfusion-weighted imaging. Magnetic Resonance in Medicine: An Official Journal of the International Society for Magnetic Resonance in Medicine. 2003;50(4):856–864. doi: 10.1002/mrm.10610 14523973

[5] Wittsack HJ, Wohlschläger AM, Ritzl EK, Kleiser R, Cohnen M, Seitz RJ, et al. CT-perfusion imaging of the human brain: advanced deconvolution analysis using circulant singular value decomposition. Computerized Medical Imaging and Graphics. 2008;32(1):67–77. doi: 10.1016/j.compmedimag.2007.09.004 18029143

[6] Mangla R, Ekhom S, Jahromi BS, Almast J, Mangla M, Westesson PL. CT perfusion in acute stroke: know the mimics, potential pitfalls, artifacts, and technical errors. Emergency radiology. 2014;21(1):49–65. doi: 10.1007/s10140-013-1125-9 23771605

[7] Potter CA, Vagal AS, Goyal M, Nunez DB, Leslie-Mazwi TM, Lev MH. CT for treatment selection in acute ischemic stroke: a code stroke primer. Radiographics. 2019;39(6):1717–1738. doi: 10.1148/rg.2019190142 31589578

[8] Vagal A, Wintermark M, Nael K, Bivard A, Parsons M, Grossman AW, et al. Automated CT perfusion imaging for acute ischemic stroke: pearls and pitfalls for real-world use. Neurology. 2019;93(20):888–898. doi: 10.1212/WNL.0000000000008481 31636160

[9] Chung CY, Hu R, Peterson RB, Allen JW. Automated Processing of Head CT Perfusion Imaging for Ischemic Stroke Triage: A Practical Guide to Quality Assurance and Interpretation. American Journal of Roentgenology. 2021;217(6):1401–1416. doi: 10.2214/AJR.21.26139 34259036

[10] Campbell BC, Christensen S, Levi CR, Desmond PM, Donnan GA, Davis SM, et al. Cerebral blood flow is the optimal CT perfusion parameter for assessing infarct core. Stroke. 2011;42(12):3435–3440. doi: 10.1161/STROKEAHA.111.618355 21980202

[11] Kamalian S, Kamalian S, Konstas A, Maas M, Payabvash S, Pomerantz S, et al. CT perfusion mean transit time maps optimally distinguish benign oligemia from true “at-risk” ischemic penumbra, but thresholds vary by postprocessing technique. American journal of neuroradiology. 2012;33(3):545–549. doi: 10.3174/ajnr.A2809 22194372 PMC3746025

[12] d’Esterre CD, Roversi G, Padroni M, Bernardoni A, Tamborino C, De Vito A, et al. CT perfusion cerebral blood volume does not always predict infarct core in acute ischemic stroke. Neurological Sciences. 2015;36(10):1777–1783. doi: 10.1007/s10072-015-2244-8 25981225

[13] Mikkelsen IK, Jones PS, Ribe LR, Alawneh J, Puig J, Bekke SL, et al. Biased visualization of hypoperfused tissue by computed tomography due to short imaging duration: improved classification by image down-sampling and vascular models. European Radiology. 2015;25(7):2080–2088. doi: 10.1007/s00330-015-3602-x 25894005

[14] Geuskens RR, Borst J, Lucas M, Boers AM, Berkhemer OA, Roos YB, et al. Characteristics of misclassified CT perfusion ischemic core in patients with acute ischemic stroke. PLoS One. 2015;10(11):e0141571. doi: 10.1371/journal.pone.0141571 26536226 PMC4633055

[15] Borst J, Marquering HA, Beenen LF, Berkhemer OA, Dankbaar JW, Riordan AJ, et al. Effect of extended CT perfusion acquisition time on ischemic core and penumbra volume estimation in patients with acute ischemic stroke due to a large vessel occlusion. PLoS One. 2015;10(3):e0119409. doi: 10.1371/journal.pone.0119409 25789631 PMC4366202

[16] Copen W, Deipolyi A, Schaefer P, Schwamm L, González R, Wu O. Exposing hidden truncation-related errors in acute stroke perfusion imaging. American Journal of Neuroradiology. 2015;36(4):638–645. doi: 10.3174/ajnr.A4186 25500309 PMC7964314

[17] Kasasbeh AS, Christensen S, Straka M, Mishra N, Mlynash M, Bammer R, et al. Optimal computed tomographic perfusion scan duration for assessment of acute stroke lesion volumes. Stroke. 2016;47(12):2966–2971. doi: 10.1161/STROKEAHA.116.014177 27895299 PMC5134896

[18] Christensen S, Lansberg MG. CT perfusion in acute stroke: practical guidance for implementation in clinical practice. Journal of Cerebral Blood Flow & Metabolism. 2019;39(9):1664–1668. doi: 10.1177/0271678X18805590 30346227 PMC6727131

[19] Cereda CW, Christensen S, Campbell BC, Mishra NK, Mlynash M, Levi C, et al. A benchmarking tool to evaluate computer tomography perfusion infarct core predictions against a DWI standard. Journal of Cerebral Blood Flow & Metabolism. 2016;36(10):1780–1789. doi: 10.1177/0271678X15610586 26661203 PMC5076783

[20] Hakim A, Christensen S, Winzeck S, Lansberg MG, Parsons MW, Lucas C, et al. Predicting infarct core from computed tomography perfusion in acute ischemia with machine learning: Lessons from the ISLES Challenge. Stroke. 2021;52(7):2328–2337. doi: 10.1161/STROKEAHA.120.030696 33957774 PMC8240494

[21] de la Rosa E, Sima DM, Menze B, Kirschke JS, Robben D. AIFNet: Automatic vascular function estimation for perfusion analysis using deep learning. Medical Image Analysis. 2021;74:102211. doi: 10.1016/j.media.2021.102211 34425318

[22] Lin L, Bivard A, Krishnamurthy V, Levi CR, Parsons MW. Whole-brain CT perfusion to quantify acute ischemic penumbra and core. Radiology. 2016;279(3):876–887. doi: 10.1148/radiol.2015150319 26785041

[23] Menze BH, Kelm BM, Weber MA, Bachert P, Hamprecht FA. Mimicking the human expert: pattern recognition for an automated assessment of data quality in MR spectroscopic images. Magnetic Resonance in Medicine: An Official Journal of the International Society for Magnetic Resonance in Medicine. 2008;59(6):1457–1466. doi: 10.1002/mrm.21519 18421692

[24] Kyathanahally SP, Mocioiu V, Pedrosa de Barros N, Slotboom J, Wright AJ, Julià-Sapé M, et al. Quality of clinical brain tumor MR spectra judged by humans and machine learning tools. Magnetic resonance in medicine. 2018;79(5):2500–2510. doi: 10.1002/mrm.26948 28994492

[25] Wei L, Rosen B, Vallières M, Chotchutipan T, Mierzwa M, Eisbruch A, et al. Automatic recognition and analysis of metal streak artifacts in head and neck computed tomography for radiomics modeling. Physics and imaging in radiation oncology. 2019;10:49–54. doi: 10.1016/j.phro.2019.05.001 33458268 PMC7807651

[26] Freund Y, Schapire RE. A decision-theoretic generalization of on-line learning and an application to boosting. Journal of computer and system sciences. 1997;55(1):119–139. doi: 10.1006/jcss.1997.1504

[27] Friedman JH. Greedy function approximation: a gradient boosting machine. Annals of statistics. 2001; p. 1189–1232.

[28] Pedregosa F, Varoquaux G, Gramfort A, Michel V, Thirion B, Grisel O, et al. Scikit-learn: Machine learning in Python. the Journal of machine Learning research. 2011;12:2825–2830.

[29] Robben D, Suetens P. Perfusion parameter estimation using neural networks and data augmentation. In: International MICCAI Brainlesion Workshop. Springer; 2018. p. 439–446.

[30] Kazemitabar J, Amini A, Bloniarz A, Talwalkar AS. Variable importance using decision trees. Advances in neural information processing systems. 2017;30.

[31] Nogueira RG, Jadhav AP, Haussen DC, Bonafe A, Budzik RF, Bhuva P, et al. Thrombectomy 6 to 24 hours after stroke with a mismatch between deficit and infarct. New England Journal of Medicine. 2018;378(1):11–21. doi: 10.1056/NEJMoa1706442 29129157

[32] Albers GW, Marks MP, Kemp S, Christensen S, Tsai JP, Ortega-Gutierrez S, et al. Thrombectomy for stroke at 6 to 16 hours with selection by perfusion imaging. New England Journal of Medicine. 2018;378(8):708–718. doi: 10.1056/NEJMoa1713973 29364767 PMC6590673

[33] Fieselmann A, Kowarschik M, Ganguly A, Hornegger J, Fahrig R. Deconvolution-based CT and MR brain perfusion measurement: theoretical model revisited and practical implementation details. Journal of Biomedical Imaging. 2011;2011:14. 10.1155/2011/467563 PMC3166726 21904538

[34] Kudo K, Sasaki M, Yamada K, Momoshima S, Utsunomiya H, Shirato H, et al. Differences in CT perfusion maps generated by different commercial software: quantitative analysis by using identical source data of acute stroke patients. Radiology. 2010;254(1):200–209. doi: 10.1148/radiol.254082000 20032153

[35] Winder A, d’Esterre CD, Menon BK, Fiehler J, Forkert ND. Automatic arterial input function selection in CT and MR perfusion datasets using deep convolutional neural networks. Medical Physics. 2020. doi: 10.1002/mp.14351 32583617

