# Supplementary figures and images for: Detecting CTP truncation artifacts in acute stroke imaging from the arterial input and the vascular output functions
Source: PLoS One. 2023 Mar 30;18(3):e0283610. doi: 10.1371/journal.pone.0283610 (PMC10062663; doi:10.1371/journal.pone.0283610)

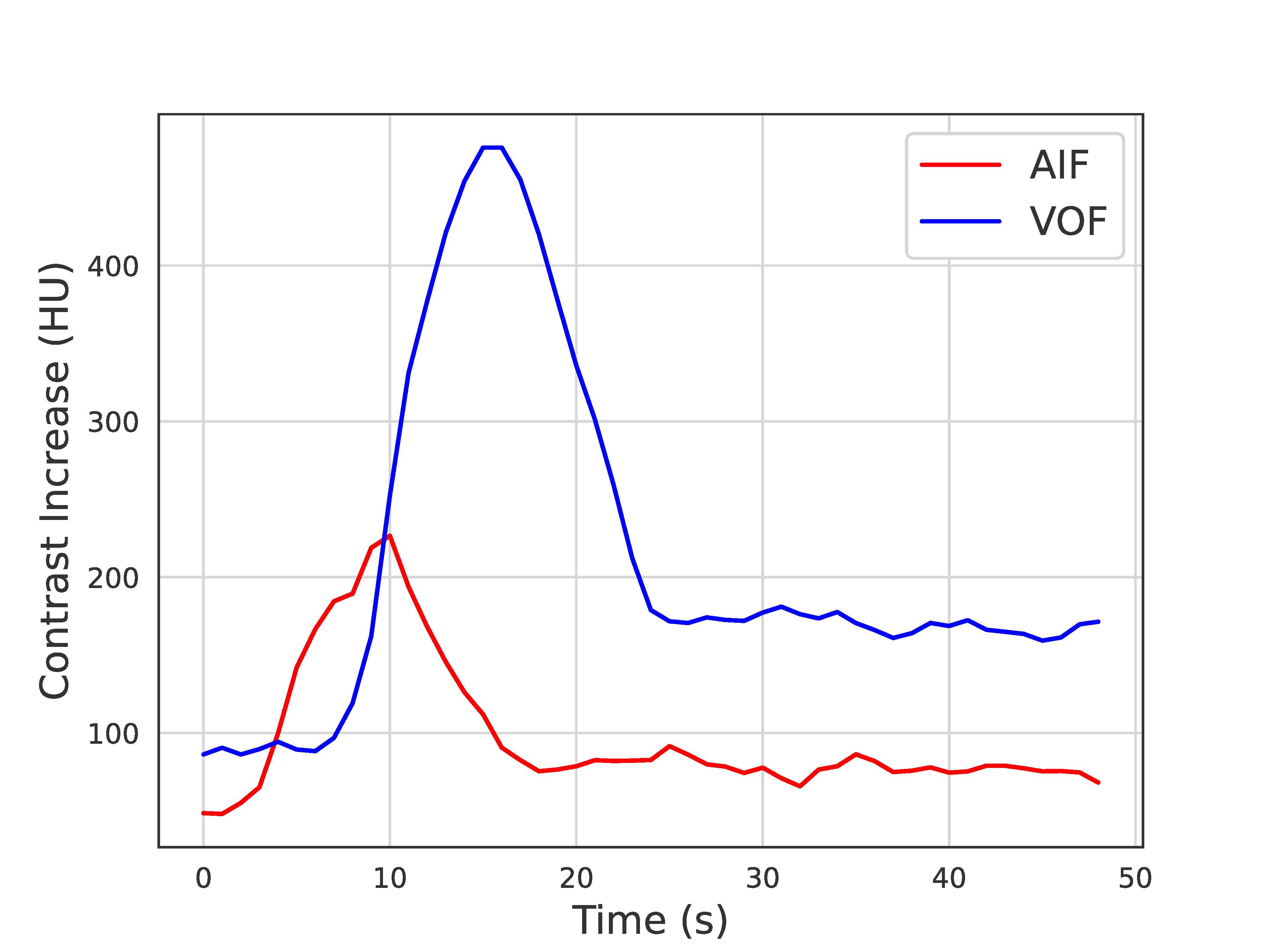

Supplement: S1 Fig — AIF: Arterial input function; VOF: Venous output function. (TIF) [file pone.0283610.s001.tif]
